# Evaluation and improvement of the regulatory inference for large co-expression networks with limited sample size

**DOI:** 10.1186/s12918-017-0440-2

**Published:** 2017-06-19

**Authors:** Wenbin Guo, Cristiane P. G. Calixto, Nikoleta Tzioutziou, Ping Lin, Robbie Waugh, John W. S. Brown, Runxuan Zhang

**Affiliations:** 1 Information and Computational Sciences, The James Hutton Institute, Invergowrie, Dundee, Scotland DD2 5DA UK; 2 Plant Sciences Division, School of Life Sciences, University of Dundee, Invergowrie, Dundee, Scotland DD2 5DA UK; 3 Division of Mathematics, University of Dundee, Nethergate, Dundee, Scotland DD1 4HN UK; 4 Cell and Molecular Sciences, The James Hutton Institute, Invergowrie, Dundee, Scotland DD2 5DA UK

**Keywords:** Gene co-expression networks, Gene regulatory networks, Network method evaluation, Partial correlation, Synthetic data

## Abstract

**Background:**

Co-expression has been widely used to identify novel regulatory relationships using high throughput measurements, such as microarray and RNA-seq data. Evaluation studies on co-expression network analysis methods mostly focus on networks of small or medium size of up to a few hundred nodes. For large networks, simulated expression data usually consist of hundreds or thousands of profiles with different perturbations or knock-outs, which is uncommon in real experiments due to their cost and the amount of work required. Thus, the performances of co-expression network analysis methods on large co-expression networks consisting of a few thousand nodes, with only a small number of profiles with a single perturbation, which more accurately reflect normal experimental conditions, are generally uncharacterized and unknown.

**Methods:**

We proposed a novel network inference methods based on Relevance Low order Partial Correlation (RLowPC). RLowPC method uses a two-step approach to select on the high-confidence edges first by reducing the search space by only picking the top ranked genes from an intial partial correlation analysis and, then computes the partial correlations in the confined search space by only removing the linear dependencies from the shared neighbours, largely ignoring the genes showing lower association.

**Results:**

We selected six co-expression-based methods with good performance in evaluation studies from the literature: Partial correlation, PCIT, ARACNE, MRNET, MRNETB and CLR. The evaluation of these methods was carried out on simulated time-series data with various network sizes ranging from 100 to 3000 nodes. Simulation results show low precision and recall for all of the above methods for large networks with a small number of expression profiles. We improved the inference significantly by refinement of the top weighted edges in the pre-inferred partial correlation networks using RLowPC. We found improved performance by partitioning large networks into smaller co-expressed modules when assessing the method performance within these modules.

**Conclusions:**

The evaluation results show that current methods suffer from low precision and recall for large co-expression networks where only a small number of profiles are available. The proposed RLowPC method effectively reduces the indirect edges predicted as regulatory relationships and increases the precision of top ranked predictions. Partitioning large networks into smaller highly co-expressed modules also helps to improve the performance of network inference methods.

The RLowPC R package for network construction, refinement and evaluation is available at GitHub: https://github.com/wyguo/RLowPC.

**Electronic supplementary material:**

The online version of this article (doi:10.1186/s12918-017-0440-2) contains supplementary material, which is available to authorized users.

## Background

Over the last fifteen years, there has been a growing interest in reverse engineering of Gene Regulatory Networks (GRNs) that aim to infer complex graphs representing transcriptional regulatory relationships, directly from gene expression profiles [1–15]. Due to its low computational complexity as well as lower requirements for the number of samples, co-expression network analysis has been widely used to infer gene regulatory networks from high throughput expression data, such as microarray or RNA-seq data [10, 16–19]. Typically thousands of genes/transcripts of special interest (e.g. differentially expressed) are utilized to construct the co-expression network in an experiment. Top candidates whose expression correlates with the gene of interest are usually further examined to identify novel regulators/targets. Despite this approach being widely used, there is a general lack of studies on the precision (the fraction of inferred regulatory relationships that are correct) and recall (the fraction of regulatory relationships that are inferred) expected.

Considerable effort has been made to evaluate the performance and robustness of GRN inference methods. The majority of evaluations were implemented on in silico datasets simulated from reference networks with sizes up to a few hundred or 1–2000 genes. Numerous studies using a range of network sizes, time-series data and perturbations have compared different analysis methods. Results are variable in terms of the top-performing method (Summaries in Additional file 1: Table S1). A series of studies have been carried out by the Dialogue for Reverse Engineering Assessments and Methods (DREAM) project, which generates challenges and organizes contests annually. The DREAM3 challenge presents gene network inference problems based on in silico networks of sizes ranging from 10, 50 and 100 genes [20–24]. Gene expression data was simulated using these networks for the following scenarios: 1) the steady state of the unperturbed networks, as well as steady state of the network where every gene is knocked out or down; and 2) 4, 23 and 46 different time series for the size 10, 50 and 100 networks respectively, with 21 time points for each time series. For example, for the network of size 100, there are a total of 1067 gene expression profiles with different perturbations and knockout/knockdown experiments available to make the inference. The inference methods: Scan Bayesian Model Averaging (ScanBMA), Gene Network Inference with Ensemble of trees (GENIE3) and Minimum Redundancy NETworks using Backward elimination (MRNETB) were the top performers in three different studies using the DREAM4 challenge time-series data, which is composed of five perturbation experiments for size 10 networks and ten perturbation experiments for size 100 networks, each with 21 time points [24–27] (Additional file 1: Table S1). Besides the DREAM benchmark datasets, the Bayesian Network (BN), Graphical Gaussian models (GGMs) and Relevance Network (RN) methods were compared using expression simulations of 100 sample points for a size 11 network with BN and GGM performing best [12]. The Algorithm for the Reconstruction of Accurate Cellular Networks (ARACNE) method had a much better performance than BN and RN on expression data with 1000 samples simulated from size 100 networks [28] while MRNET was the top ranked method when compared to the RN, ARACNE and Context likelihood or relatedness (CLR) methods on 30 datasets with different network sizes (from 100 to 1000) and sample sizes (from 100 to 1000) [29] (Additional file 1: Table S1).

A few studies aimed to evaluate network methods on larger networks of a few thousand genes. In the DREAM5 challenge, Least Absolute Shrinkage and Selection Operator (LASSO), CLR and GENIE3 are top performers among more than 30 network inference methods on a size 1643 network with 805 simulated gene expression profiles, where a list of regulators (potential transcriptional factors) are given [30]. Ten network inference methods on size 1000 network from S. Rogers [31], size 300 and 1000 networks from SynTReN [32] and size 1565 and 2000 networks from GeneNetWeaver (GNW) [24] were assessed using simulated datasets of 1000, 800, 1000, 1565 and 2000 experiments individually. CLR, GENIE3 and MRNET were the top performers in this study [33]. Similarly, ARACNE, GeneNet, Weighted Correlation Network Analysis (WGCNA) and Sparse PArtial Correlation Estimation (SPACE) were compared using size 17, 44, 83, 231, 612 and 1344 networks over datasets with 20, 50, 100, 200, 500 and 1000 sample points simulated from Gaussian distribution [34]. GeneNet ranked in the first place followed by ARACNE (Additional file 1: Table S1).

Despite the large number of evaluation studies, none have explored the normal experimental situation where a regulatory network is generated which involves hundreds and thousands of genes with only a small number of profiles being available. The assessments in the literature were based on either small and medium sized networks or datasets with a large number of samples. The evaluation conclusions were also based on a large amount of simulated expression profiles which would be difficult to validate experimentally due to the prohibitive cost or the amount of work in real experiments [35, 36].

**Fig. 1:**
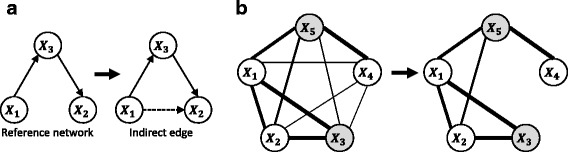
Indirect edge and RLowPC network. **a** An indirect association from *X*_1_ → *X*_2_ could arise from a regulatory structure of *X*_1_ → *X*_3_ → *X*_2_. **b** RLowPC network inference. In an RLowPC network, firstly only the top ranked edges are kept in a pre-inferred PC network and then for each pair of genes, only the immediate neighbours will be regressed for PC calculation. In this example only the top 6 of 10 edges with highest correlations are kept and PC between *X*_1_ and *X*_2_ is re-calculated by removing the effects from two immediate neighbouring nodes (*X*_3_, *X*_5_). The correlation values are represented by the thickness of the edges

Distinguishing direct regulatory interactions from indirect associations has been one of the major challenges in gene regulatory network constructions [2, 21] (see Fig. 1a). Partial Correlation (PC) is one of the methods used as a solution to distinguish direct from indirect edges of each pair of candidates by calculating the correlations after removing the linear dependencies from the remaining genes (see Fig. 1b). Other methods dealing with indirect connections include Partial Correlation coefficient with Information Theory (PCIT), ARACNE, MRNET and MRNETB. PCIT and ARACNE use the Information Theory of Data Processing Inequality method to remove the weakest gene association in each possible triplet structure in a network [37]. PCIT uses first order PC (removing the linear dependencies from the third gene in each possible triplet) to measure the significance of edge associations [38], whilst ARACNE uses Mutual Information (MI) to measure the associations between any two edges in each possible triplet [28]. MRNET uses a minimum redundancy feature selection method [39], where for each candidate gene in a MI network, it selects a subset of its highly relevant genes while minimising the MI between the selected genes [29]. MRNETB is an improved version of MRNET using a backward selection strategy starting from assuming that all genes are connected to the candidates. Less relevant genes are eliminated until the difference between the MI between a candidate and its neighbours and the MI within the neighbours are optimised [27].

Given that the search space for regulatory relationships expands factorially with the number of genes included in the network, the precision and recall of regulatory inference decrease with the increase of the network size. As gene clusters with highly cohesive patterns give rise to high correlations between all pairs of the genes in that cluster, the top ranked highly co-expressed genes may also be prone to errors of indirect associations. Here, we have developed a new method named Relevance Low order Partial Correlation (RLowPC), which is a refinement of top inferred edges by Partial Correlation methods. RLowPC selects top ranked edges from an inferred PC network as a reduced search space for indirect edges. We evaluated RLowPC alongside PC, PCIT, ARACNE, MRNET, MRNETB, and CLR on simulated time-series data and the summaries of the evaluated network inference methods is shown in Table 1. Precision and Area Under Precision-Recall curves (AUPR) were used as metrics to show that RLowPC outperforms the other methods.

**Table 1:** Summaries of the evaluated network inference methods

Category	Methods	Cor-based	MI-based	Ref.
Deal with indirect edges explicitly	RLowPC	Yes		
	PC	Yes		[2, 45]
	PCIT	Yes	Yes	[33, 38, 50, 51]
	MRNET		Yes	[29, 33, 39, 50]
	MRNETB		Yes	[27, 29, 33, 50]
	ARACNE		Yes	[28, 33, 50]
Not deal with indirect edges	Cor	Yes		
	CLR		Yes	[33, 48, 50]
	Random			

Nine correlation-based, MI-based and random network inference methods have been compared and evaluated in this study. The methods are classified into two main groups: Deal with indirect edges explicitly and Not deal with indirect edges

## Methods

### Relevance low order partial correlation (RLowPC)

The conventional pair-wise PC measures correlations after linear dependencies on all the remaining genes are removed, the majority of which may not connect to the candidates, especially in large networks where the majority of the genes only have few linked neighbours [40, 41]. Low order partial correlation methods have been proposed and utilized in the past to reduce computational complexity without much sacrifice in prediction accuracy. For example, de la Fuente et al. [42] proposed to calculate up to second order partial correlations regressing against all the remaining genes. This method was improved by confining the second order partial correlation calculation only in cases where both zero and one order PC are non-zero [43]. Our proposed RLowPC method, firstly, reduces the search space by only picking the top ranked genes from partial correlation analysis and, secondly, computes the PC by only removing the linear dependencies from the shared neighbours in the confined search space, largely ignoring the genes showing lower association and which are less relevant in the pair-wise PC calculation. The implementation details are shown below:
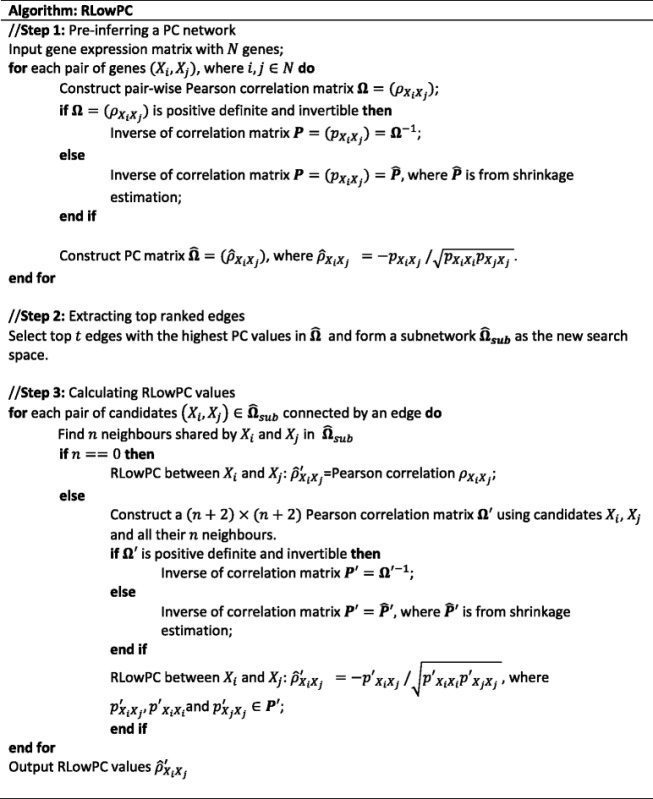


For PC and shrinkage PC calculation we have used ppcor R package [44] and corpcor R package [45], respectively.

### Gene expression data simulation

The main purpose of this study is to evaluate the performance of different network inference methods on datasets that reflect real experimental setup: large number of genes in the network with limited sample sizes and perturbations. Here, to evaluate the proposed methods comprehensively, large scale gene expression datasets were generated based on a variety of network structures using GNW version 3.1 [22, 24]. We used in silico size 100 networks in DREAM4, extracted size 500 and 1000 networks from a source *E.coli* network with 1565 nodes and 3758 edges and size 2000 and 3000 networks from a Yeast source network with 4441 nodes and 12,873 edges as reference networks. The source networks were provided by GNW [22, 24]. The networks were denoted as GNW100, GNW500, GNW1000, GNW2000 and GNW3000. Summaries for data generation can be found in Table 2. For each size, network extraction was repeated five times yielding five networks with different structures and kinetics for statistical analysis of the results. To generate the time-series, transcription kinetic models of reference networks were firstly generated in GNW by removing self-regulatory interactions and randomly assigning transcription factor (TF) genes to groups to produce protein binding complexes. In the time-series simulation procedure, Stochastic Differential Equations (SDEs) were used to model the transcription kinetics, gene activation by protein complexes, gene perturbations, mRNA and protein production and degradation. One-third of the genes in each time-series were randomly selected and perturbed from steady state at the initial time-point. Perturbations were implemented by varying the activation strengths in the protein binding simulations to enhance or inhibit the downstream expression of target genes. The perturbations were sustained until the middle of the time-series at time point 11 when the activation strengths were changed back to initial levels. A random noise term proportional to production and degradation was introduced in the SDE model, inducing high noise for activated genes and low noise for inactivated genes. The coefficient to control the noise amplitude was set to 0.05. Another random noise, which was independent to the noise in SDEs, was added at the final step to the expression data to simulate technical variations [46]. The parameters for activation strengths, production, degradation and noises were set as defaults in GNW. The time-series generation were repeated five times yielding five different time-series with different initial conditions and perturbations. Average results obtained from these time series as well as five different network structures are reported in this study. Parameter setting details are shown in Additional file 1: Figure S3 and Additional file 2: Configuration file for GeneNetWeaver. Three biological replicates were generated for each time-series. By using the replicates, analysis of variance was carried out to select genes with significant expression changes across all 21 time-points with *p*-value cut-off of 0.001. In each experiment, there are only 63 gene expression profiles generated from one perturbation used for the network construction. The repeated generation of time series data as well as the network extraction are only used for statistical purposes to take the average and calculate the variations.

**Table 2:** Source network structures and synthetic datasets

Network name		TF-gene networks	Gene No.	Edge No.	Network density	Data generator	Data type	Ref.
GNW100	GNW100_1	DREAM4 in Silico size 100	100	176	0.0356	The TF-gene reference networks were subsets of source networks in GNW. In each dataset, 1/3 genes were randomly selected and perturbed. Each experiment was sampled at 21 time points. 3 replicates were generated by adding different amount of noises. The noises are simulated by GNW. All the parameter settings were defaults in GNW.	Time-series data with multifactorial perturbation	[22, 24]
	GNW100_2		100	249	0.0503			
	GNW100_3		100	195	0.0394			
	GNW100_4		100	211	0.0426			
	GNW100_5		100	193	0.0390			
GNW500	GNW500_1	*E.coli*	500	1365	0.0109			
	GNW500_2		500	867	0.0069			
	GNW500_3		500	1107	0.0089			
	GNW500_4		500	947	0.0076			
	GNW500_5		500	1272	0.0102			
GNW1000	GNW1000_1	*E.coli*	1000	2337	0.0047			
	GNW1000_2		1000	2455	0.0049			
	GNW1000_3		1000	2089	0.0042			
	GNW1000_4		1000	2171	0.0043			
	GNW1000_5		1000	2249	0.0045			
GNW2000	GNW2000_1	Yeast	2000	4738	0.0024			
	GNW2000_2		2000	4467	0.0022			
	GNW2000_3		2000	5055	0.0025			
	GNW2000_4		2000	5283	0.0026			
	GNW2000_5		2000	4817	0.0024			
GNW3000	GNW3000_1	Yeast	3000	7515	0.0017			
	GNW3000_2		3000	7998	0.0018			
	GNW3000_3		3000	7626	0.0017			
	GNW3000_4		3000	8075	0.0018			
	GNW3000_5		3000	7333	0.0016			

A number of directed network structures were generated from source networks provided by GNW. The network names, gene and edge numbers for each structure are listed in the table. Network density is defined as the true edges divided by all possible edges. The network structures were used to simulate the time-series datasets using GNW

### Evaluation of the network inference methods

Besides the methods mentioned earlier, we also included Pearson correlation, which has been the most commonly used method to identify correlated gene pairs, as well as random guessed network, which serves a baseline for network inference performances. We also included the CLR method, which although not partial correlation-based, has been shown to perform well in several studies [30, 33, 47–49]. We divided the methods under investigation into two groups. Group one includes all the methods that deal with indirect edges explicitly, which are RLowPC, PC, PCIT, ARACNE, MRNET and MRNETB. Group two are the methods which do not deal with indirect edges explicitly and they are CLR, Pearson correlation and random guessed networks. For MI-based methods, such as ARACNE, MRNET, MRNETB and CLR networks, we have used the minet R package with default parameters [50]. The MI matrices of the methods were approximated using Pearson correlation directly from continuous time-series data [27, 49]. The PC matrices were calculated by a shrinkage approach using corpcor R package [45]. The Boolean PCIT adjacency matrices were calculated using PCIT R package [38, 51], which was used as a weight to Pearson correlation networks [33]. For the RLowPC method, the top (1500, 2000, 3000, 5000, 8000) weighted edges of inferred PC networks in GNW100, GNW500, GNW1000, GNW2000 and GNW3000 datasets were selected as search space for indirect edges. Details for tools used in the network inference analyses can be found in Table S2 in Additional file 1. In each inferred network, the top 1000 edge predictions was used to calculate True Positive (TP), False Positive (FP), True Negative (TN) and False Negative (FN) by comparing to the reference networks. The precision (TP/(TP + FP)) and pAUPR (partial plot of Area Under Precision against Recall = TP/(TP + FN)) values were calculated by picking the top ranked edges. pAUROC (partial Area Under the Receiver-Operating curve) was also calculated and the results were shown in the Supplementary material. All the evaluation of network inference methods was based on undirected network structures and the self-regulation edges were removed.

## Results

### RLowPC significantly improves the precision and recall in top predictions

**Fig. 2:**
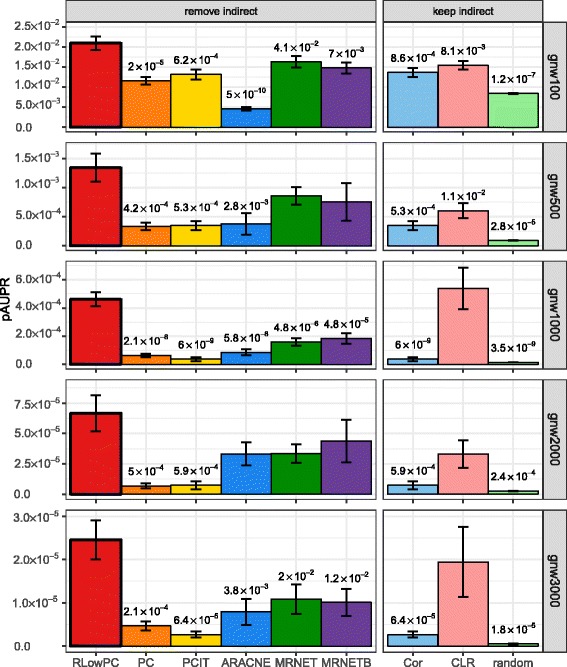
Comparison of pAUPR values for different methods and different network structures. Each bar in the plots represents mean of pAUPR values from the top 1000 edge predictions. Error bars represent standard error. The differences of pAUPR values between different methods were determined using a Student t-test in pairs between RLowPC and the other eight methods. *P*-values are shown on the top of the bars if it is less than 0.05

Figure 2 illustrates the average pAUPR values, which are the partial Area Under Precision against Recall of the top 1000 predictions, for the different methods for different network sizes. Firstly, all methods except one case for ARACNE, outperformed the random guessed network, which proves the utility of such co-expression network analysis methods. Secondly, the performances of all methods are quite consistent across different network sizes. Within Group One, RLowPC consistently performs better than all of the other methods, with MRNET/MRNETB being the next best. Within Group two, CLR clearly outperforms the most commonly employed Pearson correlation method. The differences of pAUPR values between different methods were determined using a Student t-test in pairs between RLowPC and the other eight methods (Fig. 2). Results show that the RLowPC method is able to improve the pAUPR among the top edges significantly compared to other methods except for a few cases. The pAUROC show similar results (Additional file 1: Figure S1).

**Fig. 3:**
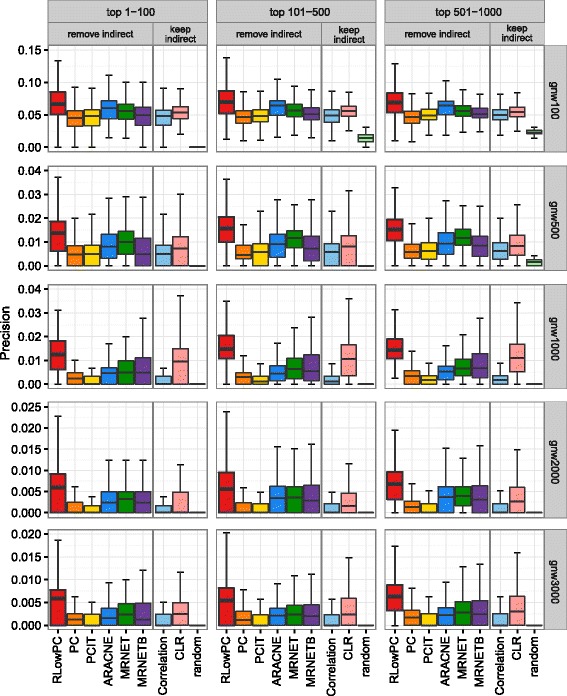
Precisions within different groups of the top 1000 predicted edges. The top 1000 predicted edges are divided into three groups, top 1–100, 101–500 and 501–1000. Each bin depicts the precision distribution of the method matched to the group and the network structures

We further divided the top 1000 predictions into groups of top 1–100, 101–500 and 501–1000 (Fig. 3). The plots indicate that, once again, the precision of RLowPC method outperformed all others, regardless of which group within the top 1000 genes were selected for investigation. MRNET, MRNETB and CLR again showed slightly better performance than PC, PCIT and ARACNE and correlation methods. It is noteworthy that the precisions of all the methods are extremely low in large networks. For example, the precision median of RLowPC in the GNW3000 networks is around 0.006, which indicates that in the top 100 predictions, only 0.6 (0.6%) edges are true predictions.

### Clustering before network inference could improve the precision and recall in top predictions

**Fig. 4:**
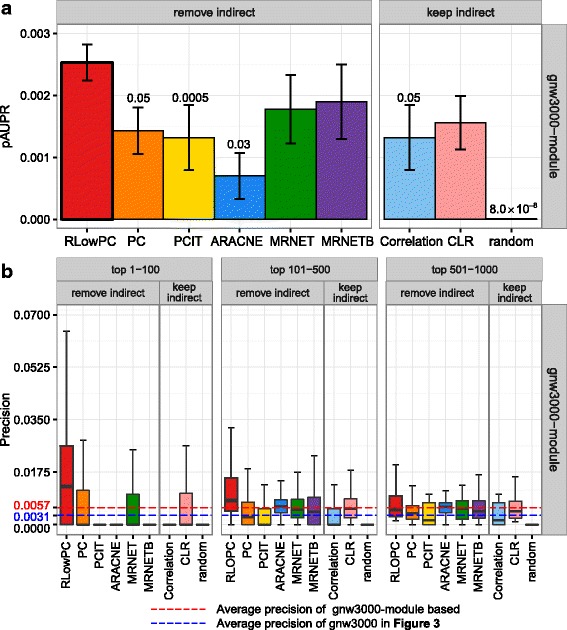
Evaluation of network analysis methods within co-expression modules by WGCNA on GNW3000 networks **a** Barplots of average pAUPR for different methods. Error bars represent standard errors of the pAUPR values across the top 1000 predictions. A Student t-test was carried out to determine the significance of the difference of pAUPR values between RLowPC and the other eight methods. *P*-values are shown on the top of the bars if it is less than 0. **b** Box plots of precisions in different groups of top 1000 edge predictions. The means of precision within modules by WGCNA (0.0057) and before clustering using WGCNA (0.0031) are shown as red and blue dashed lines

Given that precision and recall is very low among the top predictions for all methods for large networks, we explored whether precision can be improved by dividing the large networks into smaller highly cohesive clusters. Using the time-series data generated for GNW3000 as described above, all genes were clustered into non-overlapping co-expressed modules using the R package Weighted Correlation Network Analysis (WGCNA) with default settings [52, 53]. Then, network inference and evaluation were carried out separately and individually in each module. Essentially, WGCNA was used to break a big network into smaller non-overlapping subnetworks, at which point we carried out the network inference and evaluations within these smaller networks with the same time-series data. The pAUPR values were averaged across all the modules and it did not include genes that do not fit in any module (grey module). Similar to the simulation settings above, the clustering and evaluation procedures were repeated for five network structures, where five different time-series data were simulated for each structure. The average results were obtained. The average pAUPR values and precision distribution of the top 1000 predictions are presented in Fig. 4. Compared with the results of GNW3000 in Figs. 2 and 3, all methods evaluated have improved when the WGCNA method was used. This can be seen with the scale of average pAUPR values which increased from 1.0 × 10^−5^ to 1.0 × 10^−3^ (Fig. 4a), while the average precision of the top 1000 predictions has changed from 3.1 × 10^−3^ to 5.7 × 10^−3^ when the WGCNA method is used (Fig. 4b). The pAUPR value of RLowPC method is again significantly better than PC, PCIT, ARACNE, correlation and random networks. In the groups of top 1–100 and 101–500, the precision of RLowPC is better than the other eight methods and in top 501–1000 it is only better than PC, PCIT, correlation and random networks. The superior performances of RLowPC when the WGCNA method is used are also observed on the pAUROC plots (Additional file 1: Figure S2).

## Discussion

The performance of different network inference methods varies according to network structures, data quantity and quality, and methodologies. The insufficiency of sampling and the high complexity of regulation kinetics prevent precise predictions of large gene regulatory networks. As a large regulatory network is often under-determined using a small number of samples, there exists multiple plausible solutions, which cannot be distinguished by the information presented in the sample. This uncertainty in the inference of gene regulatory networks has been termed in some studies as “inferability” [54, 55]. Although our study mainly focuses on the network inference methods, special attention should be paid to generate the most informative data when trying to construct the accurate and comprehensive underlying GRNs.

**Fig. 5:**
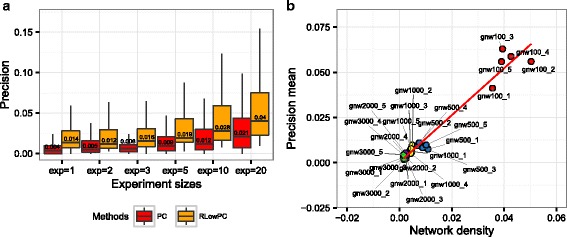
Other factors that influence the precision for network inference **a** Boxplots on the precision of PC and RLowPC methods inferred from datasets with 1, 2, 3, 5, 10 and 20 experiments with different perturbations. **b** relationships between the average precisions of all network inference methods used in this study and network density. The network names shown on the plot can be found in Table 2

The co-expression based methods capture the relationships between genes which are perturbed directly or indirectly. Therefore, the multifactorial intervention on the regulators, as discussed in [30], or hub genes rather than on target genes will generate expression data that is more informative for regulatory inference. Results presented here are based on the time-series data corresponding to one perturbation simulation to reflect more typical experimental conditions. When there are more experiments available with different sets of genes being perturbed, the inference accuracy tends to increase with the increased number of gene expression profiles available [35, 56]. Our data also show that the precision median increases as the experiment size increase (Fig. 5a). Using RLowPC, a precision of 0.014 is achieved in one experiment, while using PC on 10 experiments only leads to a precision of 0.012. Thus refining the top inferred edges using RLowPC is more effective in improving precision than generating data for nine more experiments.

With the number of possible edges growing factorially with increasing number of genes, the sparsity issue in large networks also becomes more prevalent. We observed that precision of the network inference methods increases with the increase of the network density (thus the decrease of network sparsity) as shown in Fig. 5b. Several types of methods have been explored to alleviate this problem including using network inference methods that allow imposing sparsity constraints [31, 57, 58] or leveraging on multiple datasets on other species that are evolutionary connected [59], or incorporating prior information, such as genetic maps [60], pathways, transcription factor binding, protein-protein interactions, gene ontology, epigenetics, literature, as well as functional association databases to increase the efficiency and reduce the search space by focusing on the top weighted edges [61]. RLowPC method also uses a two-step approach to select on the high-confidence edges first. Thus there is enrichment of true regulatory relationships for the second step of the inference, which explains the improvement of gene regulatory inference performances. Similarly clustering using WGCNA also groups highly correlated and connected genes together, which we see an increase of proportion in the true regulatory relationships. This has a similar effect on the network inference performances.

AUROC and AUPR curves have been popular matrices in the evaluation of network performances [21, 30, 33, 34]. AUROC measures the area under the curve between true positive rate/recall, which is calculated as (TP/(TP + FN)) and false positive rate, which is calculated as (FP/(FP + TN) = FP/N). As in big sparse networks, the negatives (N) greatly exceed the positives (P), thus false positive rate is less discriminative when the network inference methods have very different abilities to largely reduce the false positive predictions. In the meantime, AUPR measures the area under the curve between precision and recall. Precision, which is calculated as (TP/(TP + FP) = 1-FP/(TP + FP)), captures the impacts of TP or FP in the evaluation of big networks. Studies have shown that AUPR is more informative than AUROC in evaluation on datasets where the TP and TN is imbalanced. Large sparse networks are typical cases [62, 63]. As the purpose of this study is to focus on the utility of co-expression network inferences methods to prioritize the novel regulatory genes pairs for experimental validation from the top ranked edges, we mainly focused on partial AUPR curve to evaluate the accuracies and power of the network inference methods on the top weighted edges, which is more relevant than using the entire area under the curve [64, 65].

One parameter required by the RLowPC method is a number to define the search space for indirect edge reduction. For large networks, a reduction space larger than the size of the top weighted edges under investigation should be applied but has to take into account the computational search space and time required. Table 3 lists the average computational time for different sizes of search space. A useful prior may be to enrich the reduction space with true gene connections. For example, cluster analysis and functional annotation using other experimental data or regulatory databases could be carried out before network inference to investigate the functions and modules of interest.

**Table 3:** Average computational time of different sizes of reduction space using RLowPC

Top weighted edges	1500	2000	3000	5000	8000	10,000	50,000	100,000
Time	4.71	6.69	11.42	22.62	42.00	54.39	12.97	53.09
Units	secs	secs	secs	secs	secs	secs	mins	mins

The computational time is calculated based on Dell, Windows 7, 64-bit Operating system with 16.0GB RAM and Intel(R) Core (TM) i7–4790 CPU @ 3.60GHz 3.60 GHz processor

## Conclusions

In this paper, we present analysis of the evaluation of different regulatory network inference methods with special emphasis on large scale gene regulatory networks with limited sample size. We developed a new method, RLowPC, which improves the precision and recall in the top weighted PC network structures. We evaluated all methods on time-series datasets with only one perturbation for various sizes of networks using a small number of samples, which reflect better the high throughput gene expression data usually generated in laboratory experiments. We also demonstrated that clustering large co-expression networks into functional and informative co-expressed modules, improved the precision and recall of the regulatory inference.

## Additional files

Additional file 1: File contains additional Figures and Tables. **Figure S1.** Bar plots of pAUROC values for top 1000 edge predictions. **Figure S2.** Bar plots of pAUROC values of top 1000 predictions for GNW3000 module-based. **Figure S3.** GNW settings for data simulation. **Figure S4**. Examples of evaluation results. **Table S1.** Summaries of evaluation of gene network inference methods. **Table S2.** R packages used to construct and evaluate GRNs. (DOCX 1867 kb)

Additional file 2: Configuration file for GeneNetWeaver (GNW). The settings in the file were load in GNW to generate synthetic data. (DOCX 28 kb)

## Abbreviations

ARACNE: Algorithm for the reconstruction of accurate cellular networks
AUROC: Area under the receiver-operating characteristic curve
AUPR: Area under the precision recall curve
BN: Bayesian network
CLR: Context likelihood or relatedness
DREAM: Dialogue for reverse engineering assessments and methods
FN: False negative
FP: False positive
GCN: Gene co-expression network
GENIE3: Gene network inference with ensemble of trees
GNW: GeneNetWeaver
GRN: Gene regulatory network
MI: Mutual information
MRNET: Minimum redundancy networks
MRNETB: Minimum redundancy networks using backward elimination
pAUROC: Partial area under the receiver-operating characteristic curve
pAUPR: Partial area under the precision-recall curve
PC: Partial correlation
PCIT: Partial correlation coefficient with information theory
RN: Relevance network
RLowPC: Relevance low order partial correlation
SDEs: Stochastic differential eqs.
TN: True negative
TP: True positive
WGCNA: Weighted correlation network analysis
