# Supplementary material for: Evaluation and improvement of the regulatory inference for large co-expression networks with limited sample size
Source: BMC Syst Biol. 2017 Jun 19;11:62. doi: 10.1186/s12918-017-0440-2 (PMC5477119; doi:10.1186/s12918-017-0440-2)
Supplement: Supplementary file 1 — File contains additional Figures and Tables. Figure S1. Bar plots of pAUROC values for top 1000 edge predictions. Figure S2. Bar plots of pAUROC values of top 1000 predictions for GNW3000 module-based. Figure S3. GNW settings for data simulation. Figure S4. Examples of evaluation results. Table S1. Summaries of evaluation of gene network inference methods. Table S2. R packages used to construct and evaluate GRNs. (DOCX 1867 kb) [file 12918_2017_440_MOESM1_ESM.docx]

Evaluation and improvement of the regulatory inference for large co-expression networks with limited sample size

# Additional File 1

# Supplementary Figures and Tables


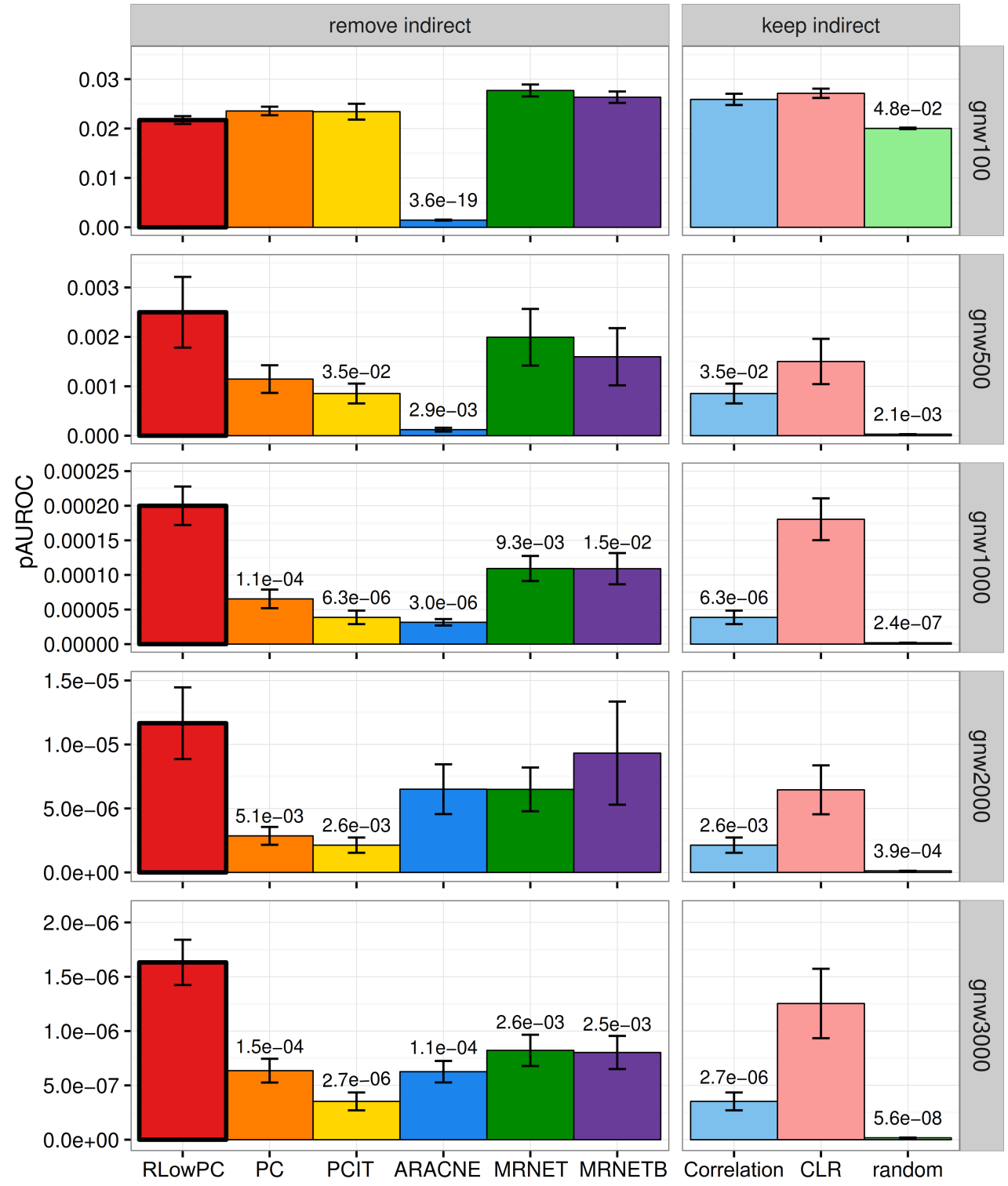


**Figure S1**. **Bar plots of pAUROC values for top 1000 edge predictions**. The plots represent the mean and standard error of pAUROC values for methods across the corresponding datasets and network structures. 100 random networks for each network structure were simulated and evaluated, shown as the last bars in the plots. The p-values (<0.05) of t-test are shown on the top of the bars.


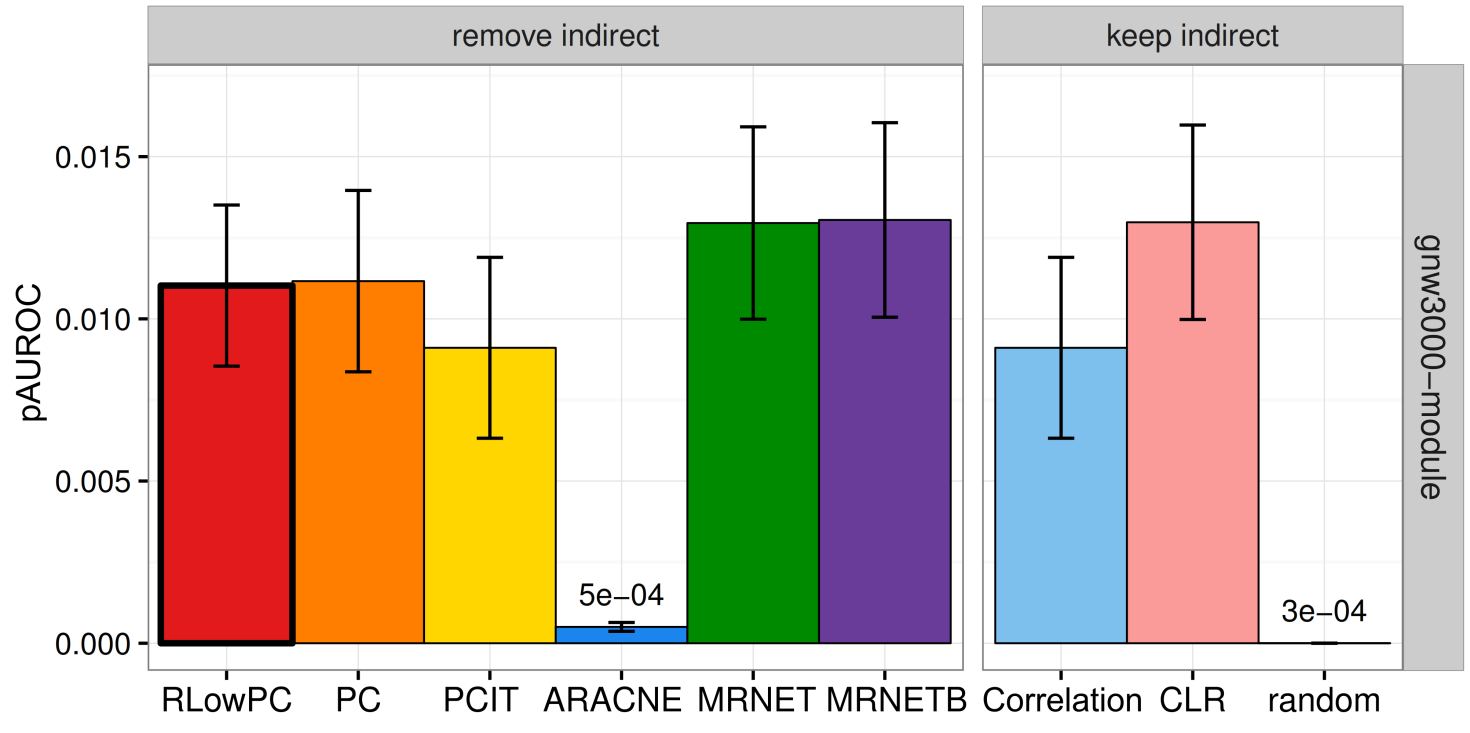


**Figure S2. Bar plots of pAUROC values of top 1000 predictions for GNW3000 module-based**. GNW3000 networks were divided into co-expressed modules using WGCNA R package [52, 53]. Network construction and evaluation were implemented in these modules. The bars in the plot indicate the pAUROC values across the methods. The mean and standard error values of the plots were calculated from all the datasets corresponding to the methods and networks. In total, 100 random networks were selected from module-based evaluation (last bins of the plots). We tested the difference of pAUROC values of RLowPC methods and methods without RLowPCor.

| **A** | 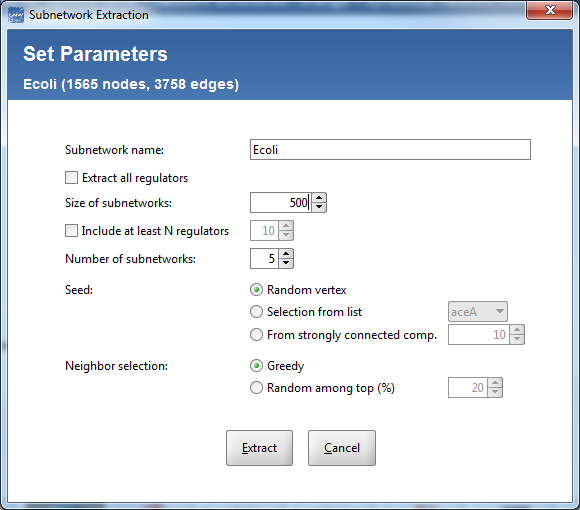 |
| --- | --- |
| **B** | **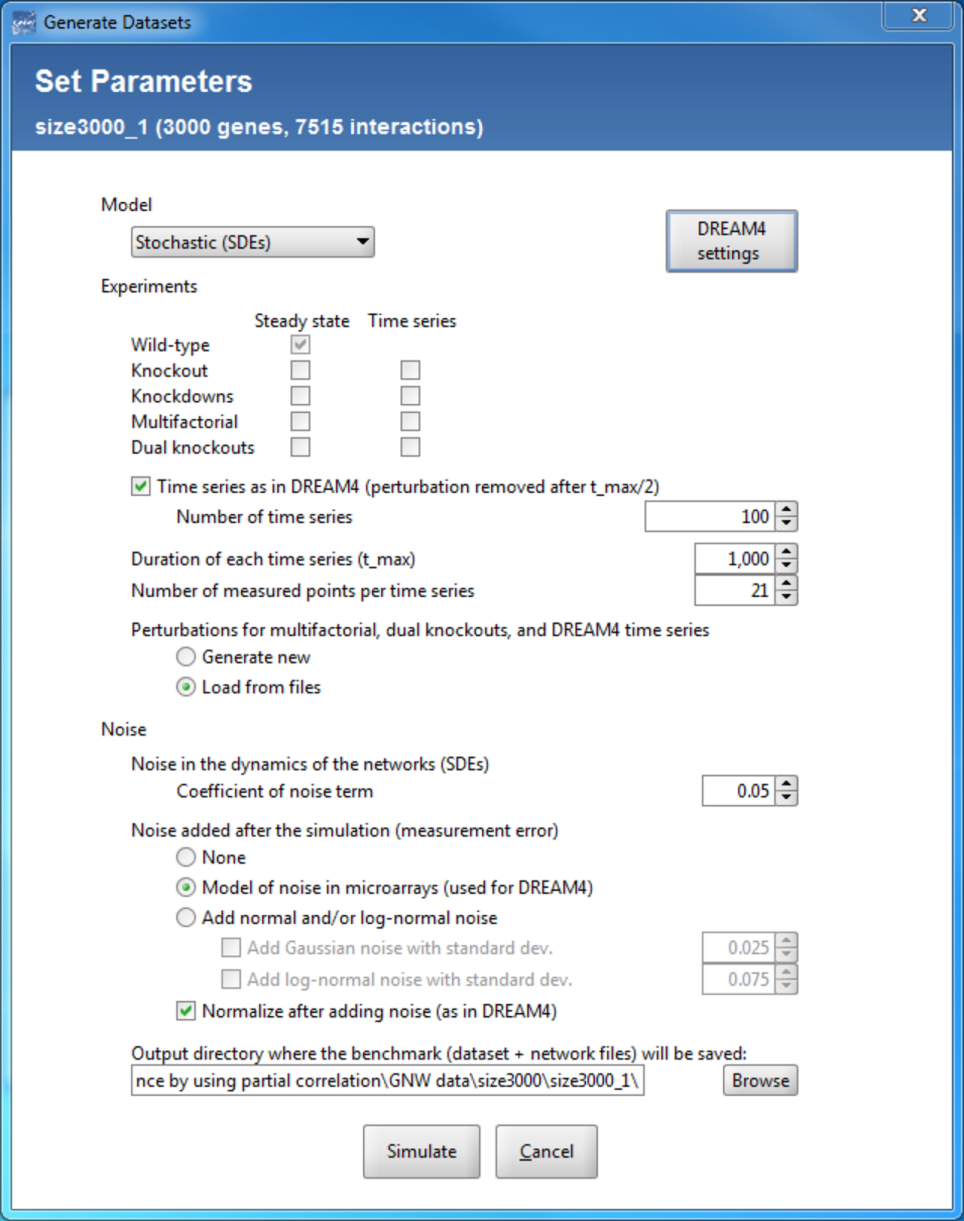** |

**Figure S3**. **GNW settings for data simulation**. (A) Subnetwork extraction panel to generate reference networks from source networks. (B) Gene expression simulation panel to generate time-series data. The settings were the same as DREAM4 challenge project [24]. Full details of settings are in Additional File 2: Configuration file for GeneNetWeaver.


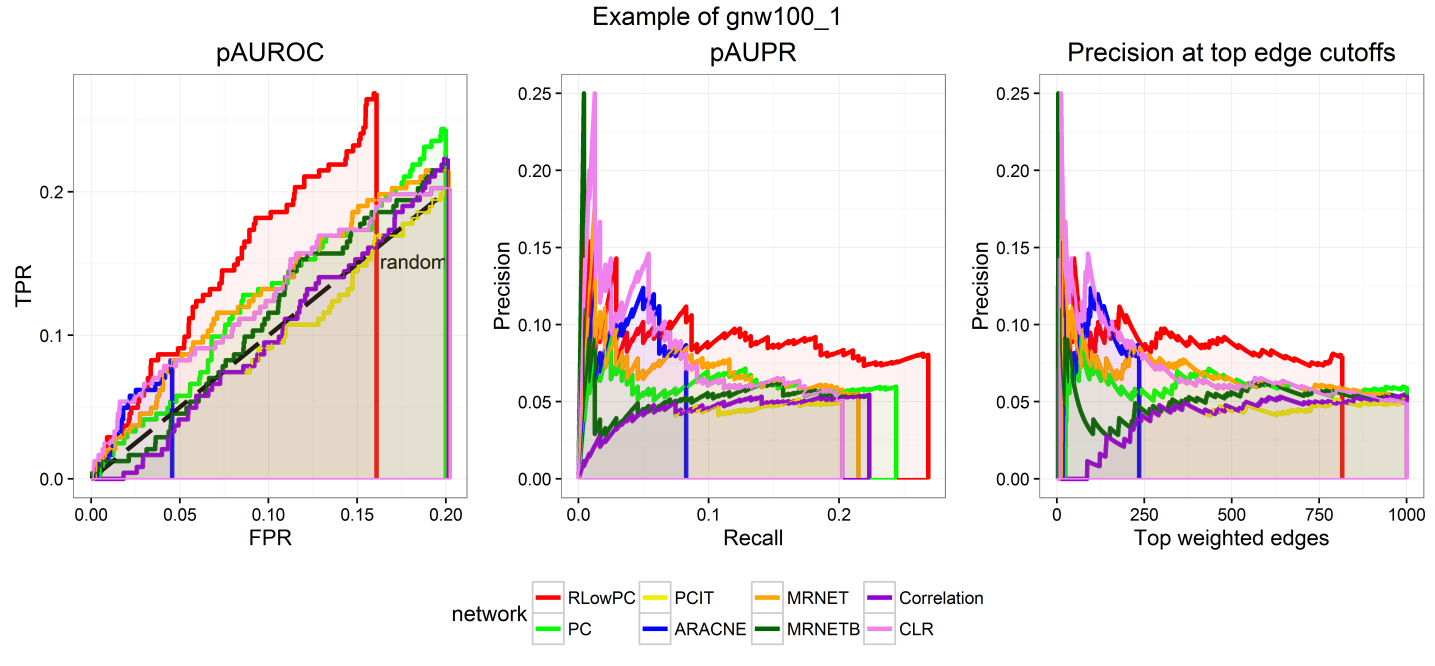


**A**


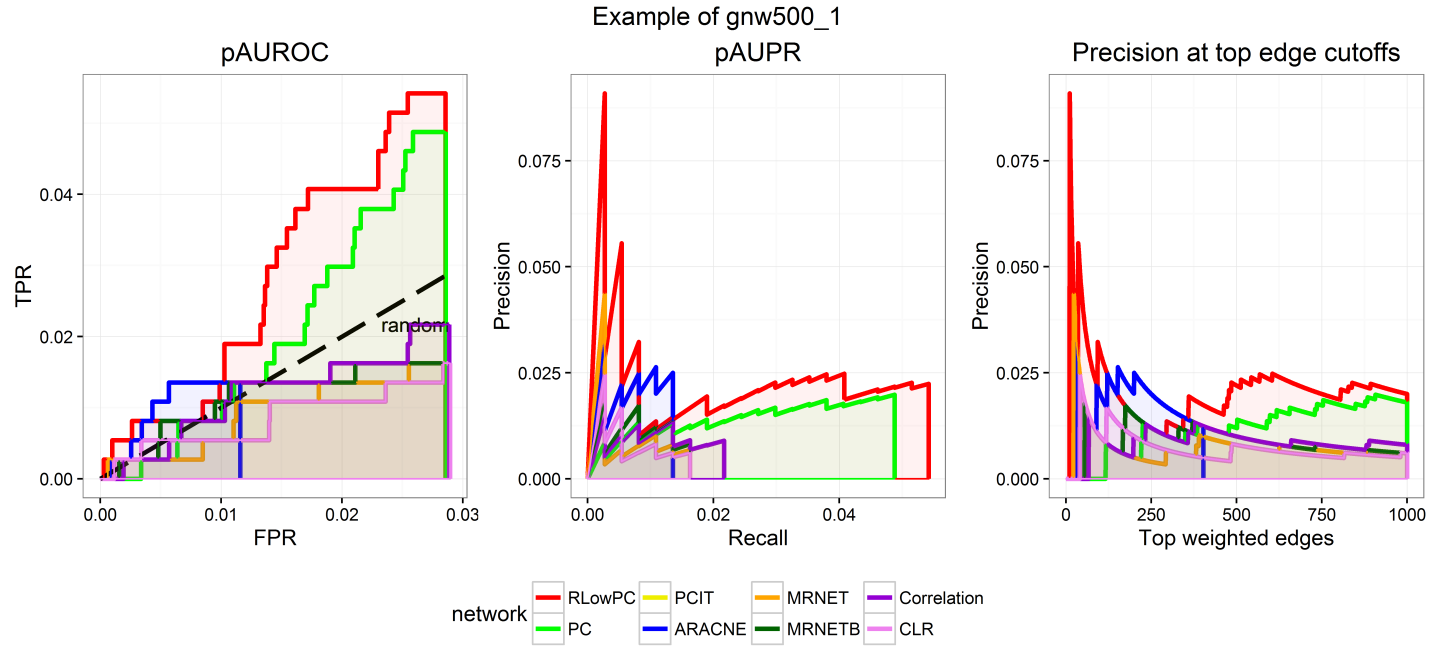


**B**


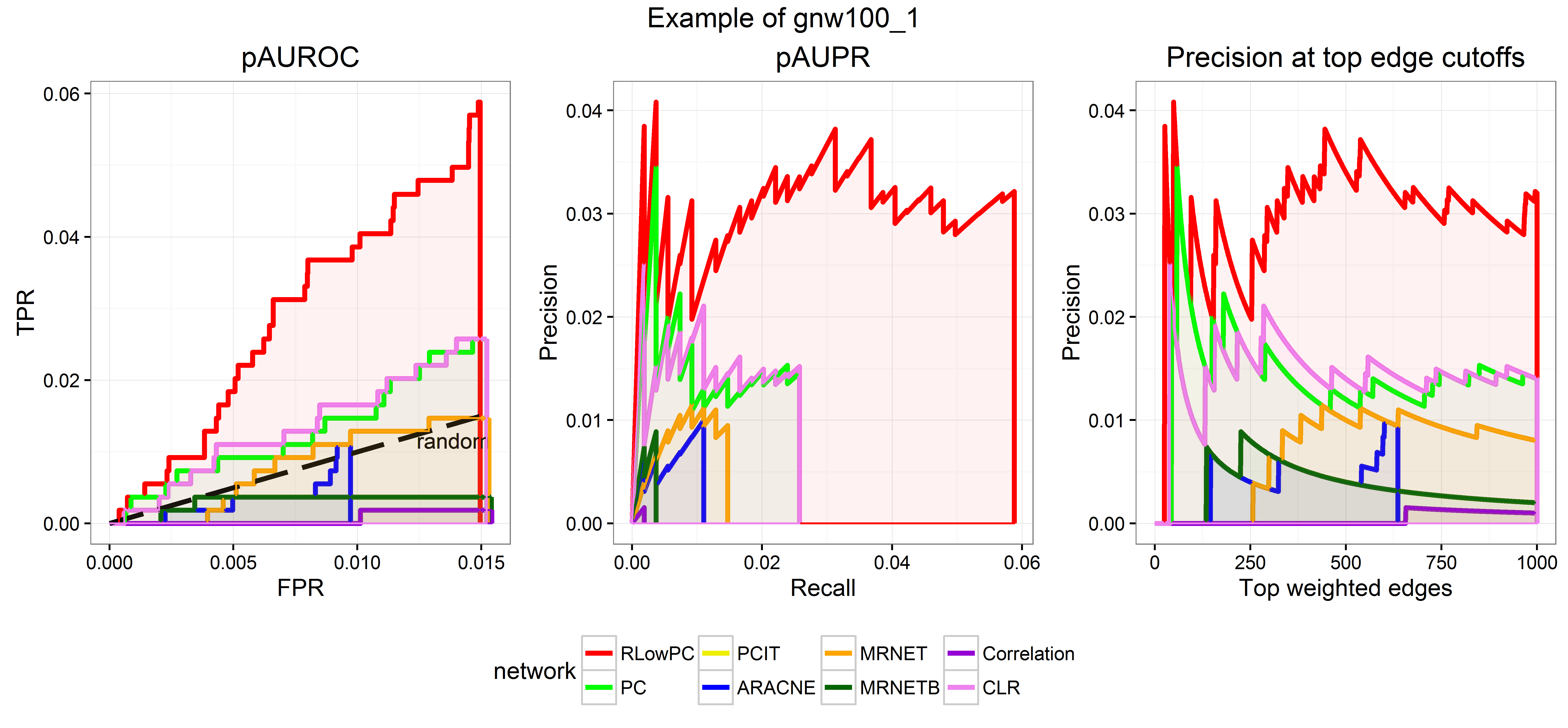


**C**


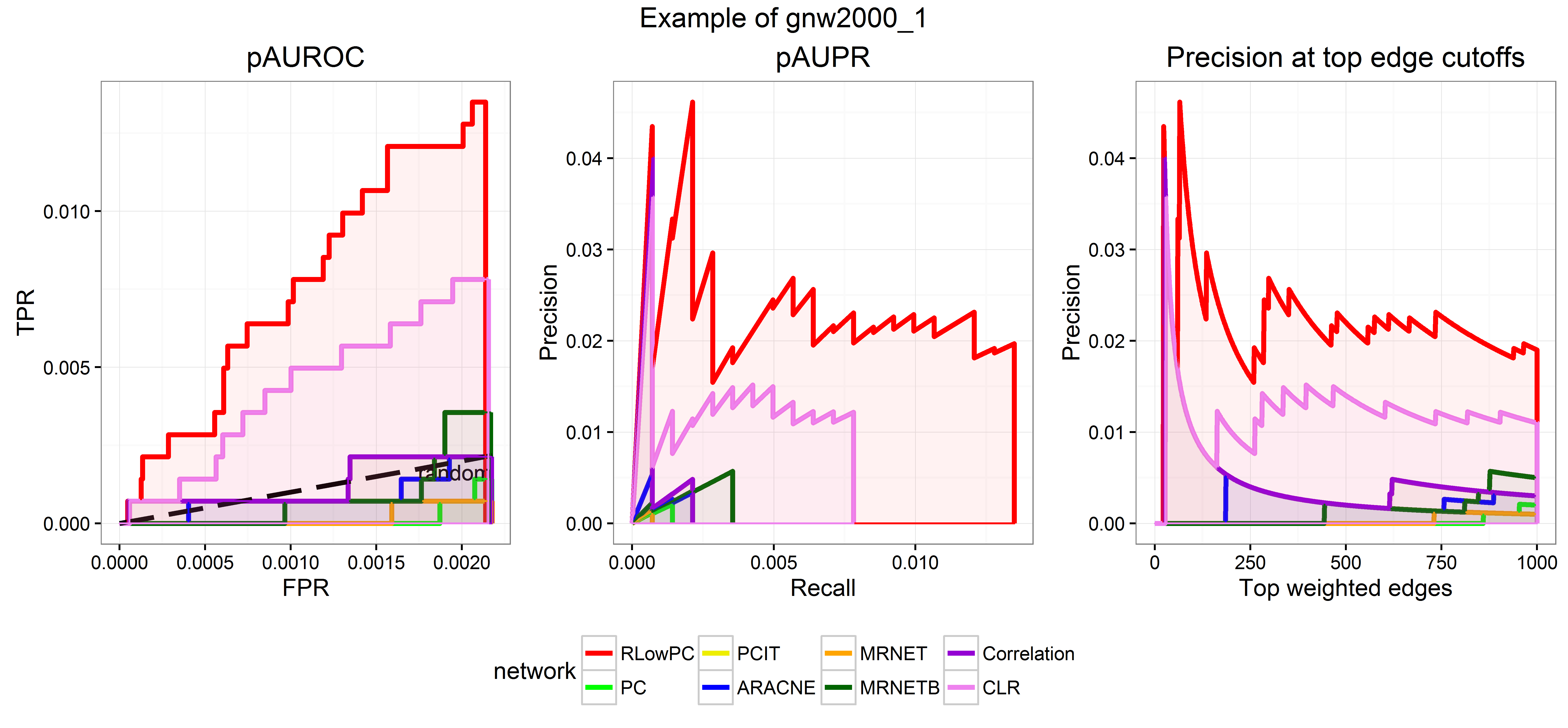

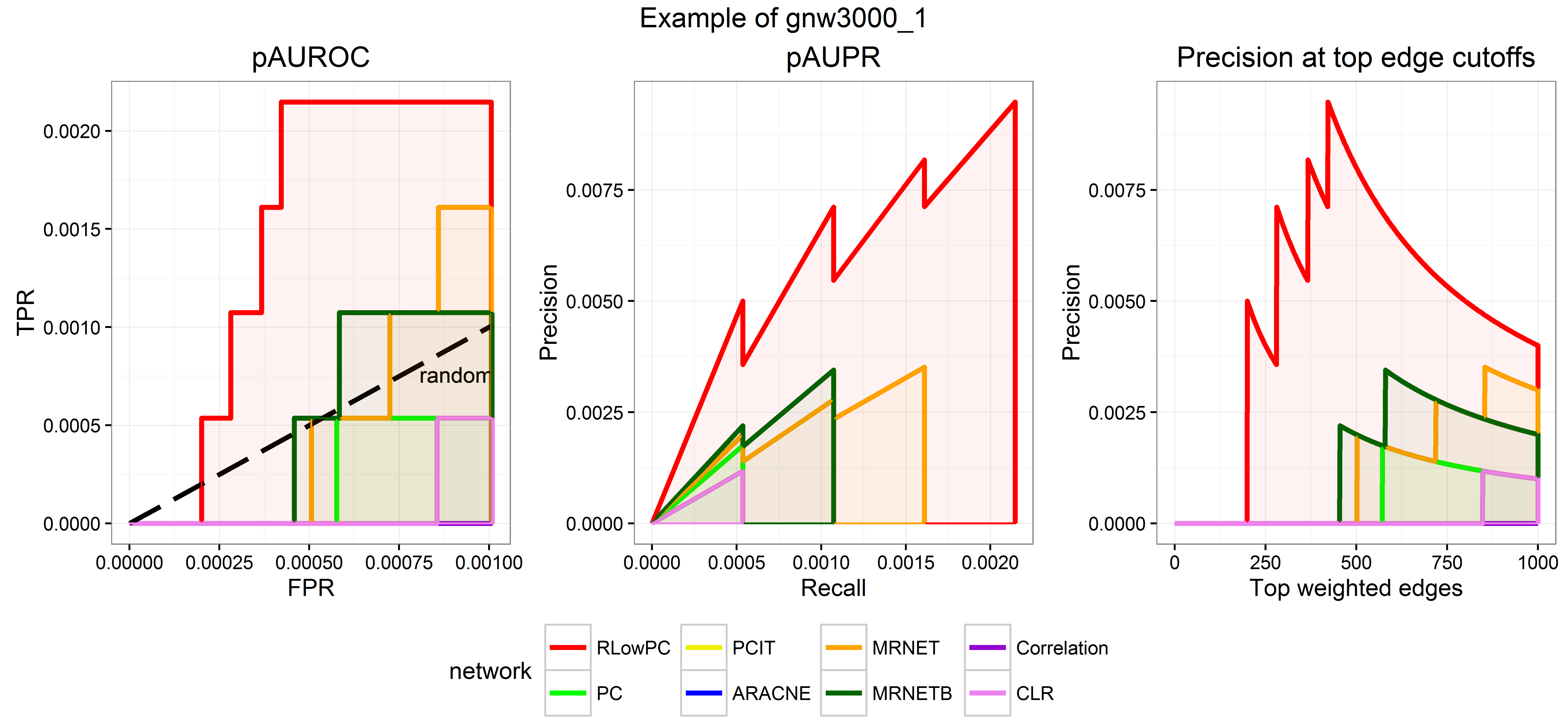


**E**

**D**

**Figure S4**. **Examples of evaluation results.** The plots show pAUROC (left), pAUPR (middle) and precision against top weighted edges (right) evaluation results of different network structures at corresponding top edge cut-offs. Each set of time-series data used to make the plots is composed of only one perturbation experiment. (A) GNW100_1 network structure, (B) GNW500_1 network structure, (C) GNW1000_1 network structure, (D) GNW2000_1 network structure and (E) GNW3000_1 network structure. In (B-E) the lines of PCIT method overlapped with correlation method since the top 1000 edge predictions for the two methods are the same. In large networks, some of the methods may not have true positive predictions at the top 1000 predictions, for example ARACNE and correlation in figure (E).

**Table S1. Summary of the evaluations of gene network inference methods**

| Scale | Project | Network size | Number of samples | Simulator | Evaluated methods | Top-ranked | Ref. |
| --- | --- | --- | --- | --- | --- | --- | --- |
| Small, median | DREAM3 | 10, 50 and 100 | Single point data: wildtype and mutant by knocking out genes one by one.  Time-series: 4, 23, and 46 perturbation experiments with 21 time points, respectively | GNW | 29 methods | Gaussian model of the noise, BN | [20-24] |
|  | DREAM4 | 10, 100 | Time-series: 5 and 10 perturbation experiments with 21 time points, respectively | GNW |  |  | [24] |
|  | ScanBMA | DREAM4 | DREAM4 | GNW | ScanBMA, LASSO, ARACNE, CLR, MRNET and iBMA | ScanBMA | [25] |
|  | GENIE3 | DREAM4 | DREAM4 | GNW | GENIE3, CLR, ARACNE, MRNET and GGMs | GENIE3 | [26] |
|  | MRNETB | DREAM4 | DREAM4 | GNW | MRNETB, CLR, ARACNE and MRNET | MRNETB, CLR | [27] |
|  | Werhli et al. [12] | 11 | Single point data: 100 sample size | Linear Gaussian distribution and non-liner ODEs | BN, GGM and RN | BN, GGM | [12] |
|  | ARACNE | 100 | Single point data: 1000 sample size | Hill kinetics | ARACNE, BN and RN | ARACNE | [28] |
|  | MRNET | 30 networks with size from 100 to 1000 | Single point data: 30 datasets with sample size from 100 to 1000 | sRogers and SynTReN | RN, ARACNE and CLR | MRNET | [29] |
| Large | DREAM5 | 1643 | Microarray simulation: 805 arrays | GNW | over 30 methods | LASSO, CLR and GENIE3 | [30] |
|  | netbenchmark | 300, 1000, 1565 and 2000 | Single point data: 20, 50, 200 and 800 sample size for each | sRogers, SynTReN and GNW | ARACNE, CLR, MRNET, MRNETB, GENIE3,Zscore PCIT, C3NET, MutRank and GeneNet | CLR, GENIE3 and MRNET | [24, 31-33] |
|  | Allen et al. [34] | 17, 44, 83, 231, 612 and 1344 | Single point data: 20, 50, 100, 200, 500 and 1000 sample size | Linear Gaussian distribution | ARACNE, GeneNet, WGCNA and SPACE | GeneNet and SPACE | [34] |

**Table S2. R packages used to construct and evaluate GRNs.**

| Package name | version | R function used | Use | Ref. |
| --- | --- | --- | --- | --- |
| RLowPC | 1.0 | RLowPC | Build RLowPC networks |  |
| PCIT | 1.5.3 | pcit | Infer PCIT network | [38, 51] |
| ppcor | 1.1 | pcor | Calculate partial correlation | [44] |
| corpcor | 1.3.8 | pcor.shrink | Calculate shrink partial correlation | [45] |
| minet | 3.28.0 | build.mim, aracne, clr, mrnet, mrnetb, validate | Build MI matrices, infer MI-based GRNs and evaluate method performance | [50] |
| WGCNA | 1.51 | pickSoftThreshold, cutreeDynamic | Partition big networks into co-expressed modules | [52, 53] |
