# Supplementary material for: Evaluation and improvement of the regulatory inference for large co-expression networks with limited sample size
Source: BMC Syst Biol. 2017 Jun 19;11:62. doi: 10.1186/s12918-017-0440-2 (PMC5477119; doi:10.1186/s12918-017-0440-2)
Supplement: Supplementary file 2 — Configuration file for GeneNetWeaver (GNW). The settings in the file were load in GNW to generate synthetic data. (DOCX 28 kb) [file 12918_2017_440_MOESM2_ESM.docx]

Evaluation and improvement of the regulatory inference for large co-expression networks with limited sample size

# Additional File 2

# Configuration file for GeneNetWeaver (GNW)

The following settings were loaded to GWN to simulate time-series gene expression datasets [24]. The details of source networks are in Table 3 main text.

#########################################################################

# Configuration file for GeneNetWeaver (GNW)

# gnw.sourceforge.net

#########################################################################

# All variables that are loaded by GnwSettings must be specified in this file, otherwise

# an Exception will be thrown and a warning message displayed.

#

# Boolean values: 0 => false, 1 => true

#========================================================================

# VARIOUS

# Seed for the random number generator, set to -1 to use current time

randomSeed = -1

# Default output directory to save files, leave empty to use the default home directory

outputDirectory = D:\\GNW data\\size3000\\size3000_1

# Model proteins and translation

modelTranslation = 1

# Set true to ignore self-links (Gi->Gi) when saving gold standards in DREAM format

ignoreAutoregulatoryInteractionsInEvaluation = 1

# Set true to explicitly list all zero interactions when saving gold standards in DREAM format (DREAM3 and DREAM4 scripts require this)

appendZeroInteractionsInGoldStandardFiles = 1

# Set true to export gene expression data also in a different format than the default (genes in rows and experiments in columns)

outputGenesInRows = 1

#========================================================================

# SUBNETWORK EXTRACTION

# The minimum number of regulators in the extracted networks, set <1 to disable control of number of regulators

numRegulators = -1

# Vertices are added using truncated selection with the given fraction (0=greedy, 1=random selection)

truncatedSelectionFraction = 0.1

# Number of seeds to be sampled from strongly connected components

numSeedsFromStronglyConnectedComponents = 0

#========================================================================

# STEADY-STATE EXPERIMENTS

# Set true (1) to generate steady states for knockouts, otherwise set false (0)

ssKnockouts = 0

# Generate steady states for knockdowns

ssKnockdowns = 0

# Generate steady states for multifactorial perturbations

ssMultifactorial = 0

# Generate steady states for perturbations as used in the DREAM4 time series

ssDREAM4TimeSeries = 0

# Generate steady states for dual knockouts

ssDualKnockouts = 0

# For deterministic simulations (ODEs), we return the steady-states as soon as convergence is reached.

# If there is no convergence until time maxtSteadyStateODE_, the values at this point are returned and a

# warning message is displayed.

maxtSteadyStateODE = 2000

# For stochastic simulations (SDEs): If maxtSteadyStateSDE < 0, we return the state at time 1.5*t_ODE,

# where t_ODE is the time of convergence for the deterministic simulation of the same perturbation.

# If maxtSteadyStateSDE > 0, we return the state at that time.

maxtSteadyStateSDE = -1

# For SDEs, every experiment starts from an independently sampled wild-type steady state. Specify here

# how long the SDEs should be simulated from the previous wild-type to get a new independent sample.

# Note, this is here in the section steady-state experiments, but it's also used for the time-series.

mintSDE = 100

#========================================================================

# TIME-SERIES EXPERIMENTS

# Set true (1) to generate time series for knockouts, otherwise set false (0)

tsKnockouts = 0

# Generate time series for knockdowns

tsKnockdowns = 0

# Generate time series for multifactorial perturbations

tsMultifactorial = 0

# Generate time series as in DREAM4

tsDREAM4TimeSeries = 1

# Generate time series for dual knockouts

tsDualKnockouts = 0

# Number of time series experiments (different perturbations are used for each time series)

numTimeSeries = 100

# Default max duration time in time-series experiments (must be consistent with numTimePoints_ and dt_)

maxtTimeSeries = 1000

# Time step for the time series (numTimePoints_ = (int)Math.round(maxtTimeSeries/dt) + 1)

dt = 50

#========================================================================

# MULTIFACTORIAL PERTURBATIONS

# Standard deviation for multifactorial perturbations

multifactorialStdev = 0.25

# The probability that a gene is perturbed (only applies for DREAM4 time series)

perturbationProbability = 0.33

# Set true to load the multifactorial perturbations from existing files

loadPerturbations = 0

# Min efficacy of gene deletions (set to 1.0 to set transcription rates to 0, set to 0.9 for 90% reduction)

minGeneDeletionEffect = 1

# Max efficacy of gene deletions (set to 1.0 to set transcription rates to 0, set to 0.9 for 90% reduction)

maxGeneDeletionEffect = 1

# Min efficacy of gene overexpressions (set to 0.5 to increase transcription rates by 50%)

minGeneOverexpressionEffect = 1

# Max efficacy of gene overexpressions (set to 1.0 to increase transcription rates by 100%)

maxGeneOverexpressionEffect = 1

# Min fraction of TFs that are direct targets of a drug perturbation

minFractionDirectTargets = 0.05

# Max fraction of TFs that are direct targets of a drug perturbation

maxFractionDirectTargets = 0.3

#========================================================================

# DETERMINISTIC MODEL (ODE)

# If set true, a deterministic simulation of the experiments is done using the ODE model

simulateODE = 0

# Absolute _or_ relative precision _per variable_ need to be satisfied for convergence

absolutePrecision = 0.00001

# See absolutePrecision_, in addition, this is also the tolerance used for integration

relativePrecision = 0.001

#========================================================================

# STOCHASTIC MODEL (SDE)

# If set true, a stochastic simulation of the experiments is done using the SDE model

simulateSDE = 1

# Time step used for integrating SDEs (internal dt used for integration, the measured points are defined by numMeasuredPoints)

timeStepSDE = 1.0

# Coefficient of the noise term of the SDEs

noiseCoefficientSDE = 0.05

#========================================================================

# EXPERIMENTAL NOISE

# Set true (1) to add normal noise to the data after simulation

addNormalNoise = 0

# Set true to add lognormal noise to the data

addLognormalNoise = 0

# Set true to use a realistic model of microarray noise, similar to a mix of normal and lognormal

addMicroarrayNoise = 1

# The standard deviation of the normal noise

normalStdev = 0.025

# The standard deviation of the lognormal noise

lognormalStdev = 0.075

# Set true to normalize the datasets after adding the experimental noise

normalizeAfterAddingNoise = 1
